# Elevated somatostatin interneuron long-term potentiation minimally regulates temporoammonic plasticity in a mouse model of Fragile X Syndrome

**DOI:** 10.3389/fphar.2025.1640921

**Published:** 2026-01-15

**Authors:** Max A. Wilson, Anna Sumera, Emre Berk, Sam A. Booker

**Affiliations:** 1 Institute for Neuroscience and Cardiovascular Research, University of Edinburgh, Edinburgh, United Kingdom; 2 Simons Initiative for the Developing Brain, University of Edinburgh, Edinburgh, United Kingdom; 3 Patrick Wild Centre, University of Edinburgh, Edinburgh, United Kingdom

**Keywords:** *Fmr1* mouse, Fragile X Syndrome, GABAB receptor, group 1 metabotropic glutamate receptor, hippocampus, somatostatin interneuron, synaptic plasticity (LTP), temporoammonic pathway

## Abstract

**Introduction:**

Fragile X Syndrome is a common, inherited single gene cause of intellectual disability, associated with autism, epilepsy, anxiety, and sensory disturbances. Many of these features have been attributed to cellular dysfunction leading to impaired synaptic plasticity, in particular through metabotropic glutamate and GABA receptor signalling. The function of these pathways in inhibitory interneurons has not been fully elucidated. In this study we test the hypothesis that somatostatin interneurons (SST-INs) display impaired synaptic plasticity, which leads to circuit-level plasticity deficits.

**Methods:**

We use a combination of whole-cell and extracellular recordings in acute hippocampal brain slices prepared from adult, male wild-type and *Fmr1* ^-/y^ mice.

**Results:**

We find that long-term potentiation in SST-INs is enhanced in *Fmr1* ^-/y^ mice, and that this plasticity is susceptible to GABA_B_ receptor activation. However, long-term potentiation at temporoammonic inputs to CA1 region is not impaired in *Fmr1* ^-/y^ mice following tetanic stimulation. We find that temporoammonic long-term potentiation is equivalently modified by metabotropic glutamate and GABA receptor pharmacology, despite changes in presynaptic function.

**Discussion:**

These data show that while SST-IN function is impaired in *Fmr1* ^-/y^ mice, circuit level plasticity is maintained. This study provides new insights into the function of drugs proposed for the treatment of Fragile X Syndrome.

## Introduction

Fragile X Syndrome (FXS) is a common and inherited cause of intellectual disability, resulting from silencing of the *FMR1* gene leading to loss of the Fragile X Messenger Ribonuclear Protein (FMRP); a RNA-binding protein that regulates protein synthesis and function (Bassell and Warren, 2008). FXS is typified in affected individuals by intellectual disability, cognitive inflexibility, seizures, autism, sensory alterations, and anxiety; many of which have a basis in altered neuronal excitability and plasticity (Booker and Kind, 2021; Contractor et al., 2015) and which have been identified in *Fmr1* ^*-/y*^ mouse and rat models (Gibson et al., 2008; Domanski et al., 2019; Booker and Kind, 2021; Asiminas et al., 2022).

Research into FXS using rodent models has been dominated by the mGluR theory (Huber et al., 2002), which proposes that exaggerated group I metabotropic glutamate receptor (mGluR) signalling, especially via mGluR5, leads to unchecked activity-dependent protein synthesis and exaggerated long-term depression (LTD.; Bear, 2005; Bear et al., 2004). Additional to the mGluR theory, loss of FMRP leads to changes in cellular excitability. Beyond modifying the excitability of excitatory neurons, evidence suggests impaired inhibitory signalling (Antoine et al., 2019; Domanski et al., 2019; Gibson et al., 2008), including loss of ionotropic GABA_A_ receptors (GABA_A_R) (d'Hulst et al., 2006; Gantois et al., 2006) and metabotropic GABA_B_ receptors (GABA_B_R) (Kang et al., 2017; Wahlstrom-Helgren and Klyachko, 2015). These synaptic and cellular abnormalities are thought to underpin many FXS-related phenotypes including sensory hypersensitivity, learning/memory impairments, and epilepsy (see Contractor et al. (2015) for review); but how GABA and glutamate receptors contribute to hippocampal circuit function remains not fully explored.

In the hippocampus, synaptic inputs onto CA1 pyramidal neurons (PNs) are organised along two distinct pathways: Schaffer collateral (SC) inputs from CA3 targeting proximal dendrites, and temporoammonic (TA) inputs from layer III of the lateral entorhinal cortex, which target distal dendrites in *stratum lacunosum-moleculare* (SLM) (Remondes and Schuman, 2002; Amaral and Witter, 1989); the latter of which is associated with contextual and spatial memory formation (Vago and Kesner, 2008). In rodent models of FXS, TA inputs to CA1 display reduced strength (Asiminas et al., 2022; Booker et al., 2020) and feed-forward inhibition in the TA pathway is weakened (Wahlstrom-Helgren and Klyachko, 2015). A major subtype of hippocampal GABAergic interneuron expressing the neuropeptide somatostatin (SST-IN) form synapses that co-align with TA inputs, while receiving predominantly feedback inputs from CA1 PNs (Booker and Vida, 2018; Booker et al., 2018; Ali and Thomson, 1998). SST-INs have been shown to regulate the action potential firing activity (Royer et al., 2012) and synaptic integration of PNs (Lovett-Barron et al., 2012), contribute to theta and beta oscillatory activity (Artinian et al., 2019; Katona et al., 2014; Vasuta et al., 2015), and gate multimodal information flow between and within cortical regions (Abbas et al., 2018; Artinian et al., 2019; Leão et al., 2012; Naka et al., 2019). Importantly for FXS, SST-INs undergo synaptic plasticity mediated by group 1 mGluRs (mGluR1α and mGluR5) (Topolnik et al., 2006), with long-term potentiation (LTP) at excitatory synapses onto SST-INs inhibiting the TA region and enhancing SC-CA1 synapses via disinhibitory connections (Le Duigou and Kullmann, 2011; Vasuta et al., 2015). Plasticity of SST-INs is inhibited by activation postsynaptic GABA_B_Rs, which directly suppress dendritic L-type Ca^2+^ channels (Booker et al., 2018). As such, pharmacological targeting of GABA_B_R and mGluR pathways may have profound effects on the SST-IN microcircuit. Indeed, a recent study examining TA LTP in *Fmr1* ^-/y^ mice revealed reduced plasticity (Ordemann et al., 2021), however this study blocked both GABA_A_R and GABA_B_Rs throughout, precluding the influence of SST-IN GABAergic signalling. Drugs targeting both GABA and mGluR signalling have undergone clinical trials in FXS, notably baclofen (a selective GABA_B_R agonist; Berry-Kravis et al., 2012), Fenobam, mavoglurant, and basimglurant (mGluR5 negative allosteric modulators and antagonists; Berry-Kravis et al., 2016; Berry-Kravis et al., 2009), and ERK signalling (lovastatin, Çaku et al., 2014). Meanwhile, compounds like ganaxolone, which target extrasynaptic GABA_A_Rs, have shown preclinical benefits by restoring inhibitory tone (Ligsay et al., 2017). Thus, GABAergic SST-INs, may be uniquely placed to modify inhibitory circuit function, contributing to impaired hippocampal processing in FXS.

We hypothesised that SST-INs in *Fmr1* ^-/y^ mice may undergo excessive LTP, through mGluR1/5 dependent mechanisms, leading to impaired TA-CA1 plasticity. Further, we posited that pharmacological modulation of SST-IN signalling could differentially modify circuit plasticity. Using whole-cell recordings from SST-INs in CA1 and extracellular field recordings of TA synaptic responses, we assessed the impact of drugs targeting GABA_B_Rs, GABA_A_Rs, mGluR1α, and mGluR5. We show that while LTP in SST-INs in *Fmr1* ^-/y^ mice is elevated, this does not lead to circuit level defects. Further, we show that TA-LTP in *Fmr1* ^-/y^ mice displays no differences in sensitivity to mGluR1/5 or GABA receptor pharmacology.

## Materials and methods

### Animals

All procedures were performed according to Home Office (ASPA, 2013) and The University of Edinburgh Ethical Board guidelines. *Fmr1* ^-/y^ mice were maintained on a C57/Bl6J^CRL^ background, housed on a 12h light/dark cycle, and with *ad libitum* access to food and water. For all experiments, adult (8–18 weeks) male mice were used, due to the X-linked nature of the *Fmr1* gene. Some mice were maintained as heterozygous for Cre-recombinase under the SSt promoter. For all experiments WT and *Fmr1* ^*-/y*^ mice were used, with experiments blind to genotype during recording and analysis.

### Acute brain slice preparation

Brain slices were prepared as previously described (Oliveira et al., 2021). Briefly, mice were terminally anaesthetised with isoflurane, then decapitated and their brains rapidly dissected into ice-cold sucrose-modified artificial cerebrospinal fluid (sucrose-ACSF; ACSF; in mM: 87 NaCl, 2.5 KCl, 25 NaHCO_3_, 1.25 NaH_2_PO_4_, 25 glucose, 75 sucrose, 7 MgCl_2_, 0.5 CaCl_2_) which was saturated with carbogen (95% O_2_/5% CO_2_). Brains were glued to a vibratome stage (Leica VT1200S, Leica, Germany), then either 400 µm (for whole-cell recordings) or 500 µm (for extracellular recordings) horizontal slices containing the hippocampi were cut. Following slicing, brain slices were placed in either: a submerged holding chamber containing sucrose-ACSF warmed to 35 °C for 30 min, then at room temperature; or on small squares of filter paper placed in a liquid/gas interface chamber, containing recording ACSF (in mM: 125 NaCl, 2.5 KCl, 25 NaHCO_3_, 1.25 NaH_2_PO_4_, 25 glucose, 1 MgCl_2_, 2 CaCl_2_) and bubbled with carbogen.

### Whole-cell recording

For whole-cell recordings from SST-INs, slices were transferred to a submerged recording chamber, flowing at 6–8 mL/min with recording ACSF, which was carbogenated and warmed to near physiological temperatures (31 °C ± 1 °C) by an inline heater (Scientifica, United Kingdom). Slices were visualized with an upright microscope (SliceScope, Scientifica, United Kingdom), equipped with a ×40 water-immersion objective lens (N.A. 0.8) using a digital camera (SciCam Pro, Scientifica, United Kingdom). Whole-cell patch-clamp recordings were made using a Multiclamp 700B amplifier (Molecular Devices, United States). Recording pipettes were pulled from borosilicate glass capillaries (1.5 mm outer/0.86 mm inner diameter, Harvard Apparatus, United States) on a horizontal electrode puller (P-1000, Sutter Instruments, CA, United States). Pipettes were filled with K-gluconate based solution (in mM: 142 K-gluconate, 4 KCl, 0.5 EGTA, 10 HEPES, 2 MgCl_2_, 2 Na_2_ATP, 0.3 Na_2_GTP, 10 Na_2_Phosphocreatine, 2.7 Biocytin, pH = 7.4, 290–310 mOsm), giving a resistance of 4–6 MΩ. Unless otherwise stated, all voltage-clamp recordings were performed at a holding potential of −65 mV and all current-clamp recordings from the resting membrane potential (V_M_). Access resistance (R_A_) was monitored, but not compensated in voltage-clamp and the bridge balanced in current-clamp. Signals were filtered online at 2–10 kHz using the built in 2-pole Bessel filter of the amplifiers, digitized and acquired at 20 kHz (Digidata 1550B, Axon Instruments, United States), using pClamp 11 (Molecular Devices, CA, United States) acquisition software. Data were analyzed offline using the open source Stimfit software package (Schlögl et al., 2013).

Putative SST-INs were identified in brain slices as horizontally oriented cells at the *str. oriens*/*alveus* border, which in response to −125 to +125 pA (25 pA steps, 500 ms duration) produced a characteristic large voltage “sag” in response to hyperpolarising currents and moderate frequency, repetitive, non-adapting trains of action potentials to depolarising stimuli. LTP was induced in SST-INs in whole-cell recordings as previously described (Booker et al., 2018). EPSCs were elicited by a bipolar stimulating electrode placed in the alveus of CA1 ∼500 µm distal from the SST-IN soma. Following a 5-min control baseline, LTP was induced with a theta-burst stimulation (TBS) paradigm, which combined associative pairing of five trains of presynaptic EPSCs (4 stimuli at 100 Hz) with postsynaptic depolarization to −20 mV (60 ms duration), repeated 3 times at 30 s intervals as has been described previously (Topolnik et al., 2006). The resulting synaptic potentiation was recorded for at least 25 min following the TBS stimuli and LTP expressed as the change in mean EPSC amplitude measured between 20–25 min post-TBS relative to the 5-minute-baseline preceding the TBS. To activate GABA_B_Rs, slices were treated with 20 μM baclofen for 20 min prior to LTP induction. Following recordings, all cells were sealed with outside-out patch configuration, then fixed for 24–72 h in 4% paraformaldehyde in 0.1 M phosphate buffer (PB).

### Visualization, imaging and reconstruction of the recorded neurons

Post hoc identification of recorded neurons was performed as previously described (Booker et al., 2014). Slices were rinsed in phosphate buffered saline (PBS; 0.1 M PB + 0.9% NaCl) and then blocked with 10% normal goat serum (NGS), 0.5% TritonX-100% and 0.05% NaN_3_ diluted in PBS for 1 h at room temperature. Slices were incubated for 72 h in a solution containing 5% NGS, 0.5% TritonX-100% and 0.05% NaN_3_ and primary antibodies against SST-14 (rabbit, 1:500, Peninsula Laboratories, United States) at 4 °C. Slices were then rinsed in PBS and then incubated with fluorescently conjugated secondary antibodies (Goat anti-rabbit IgG, AlexaFluor 568; 1:500, Invitrogen, United Kingdom) and fluorescent-conjugated streptavidin (AlexaFluor 633; 1:500, Invitrogen, United Kingdom) in a solution containing 3% NGS, 0.1% TritonX-100% and 0.05% NaN_3_ for 24 h at 4 °C. Slices were rinsed in PBS, then PB, and mounted on glass slides (Fluoromount-G, Southern Biotech, AL, United States). Biocytin filled cells were imaged with a laser scanning confocal microscope (SP8, Leica, Germany) under a 20× (N.A 0.75) objective and z-axis stacks of images (2048 × 2048 pixel radial resolution, 1 µm axial steps) collected to allow identification of somato-dendritic and axonal arborizations. To assess immunoreactivity of the recorded neurons the somata of neurons were imaged with an oil-immersion 63× (N.A 1.3) objective lens, with images taken over the somata. Example cells were reconstructed offline from 20x stacks using the SNT plug-in for the FIJI (Longair et al., 2011).

#### Extracellular field recording

For field excitatory postsynaptic potential (fEPSP) recordings, slices were transferred to an interface recording chamber perfused with carbogenated recording ACSF at 2–3 mL/min and maintained at 30 ± 1 °C. Recording pipettes with a resistance of 1–3 MΩ were pulled from borosilicate glass capillaries (1.5 mm outer/0.86 mm inner diameter, Harvard Apparatus, United Kingdom) on a horizontal electrode puller and filled with recording ACSF. Slices were visualised using a wide-field microscope (Leica, Germany) and pipettes placed in stratum lacunosum-moleculare (SLM) layer of hippocampal CA1. LTP was induced in extracellular field recordings from CA1. fEPSPs were evoked using a paired-pulse protocol via a bipolar stimulating electrode placed in SLM of CA1, ∼500 μm to 1 mm distal to the recording electrode, targeting the TA pathway. Following a 10-min baseline, pharmacological agents were applied via the circulating aCSF. To activate GABA_B_Rs, slices were treated with 20 μM baclofen for 20 min, followed by a 20 min washout. To antagonise mGluR1α and mGluR5, 20 μM LY367385 and 10 μM Fenobam were bath-applied, respectively, and responses recorded for 30–40 min. To block GABA_A_Rs, 10 μM gabazine was applied, with effects measured 20–30 min post-application. In gabazine experiments, CA3–CA1 projections were severed to prevent recurrent excitation.

A new 10 min baseline was recorded in each drug condition or following washout. LTP was induced using a high-frequency stimulation (HFS) protocol consisting of two 100 Hz trains of 100 pulses, delivered 30 s apart. Potentiation was monitored for 60 min post-HFS, and LTP magnitude expressed as the mean fEPSP slope measured between 50 and 60 min relative to the 10-min baseline. LTP was considered successful if the 50–60-min slope exceeded baseline by >10%. Signals were rejected if the average baseline slope of 1-2 and 9–10 min were ±10%. Recordings were filtered online with a 1 Hz high-pass and 500 Hz low-pass filter and digitised at 10 kHz. All data were acquired and analysed offline using WinLTP (v3.01, University of Bristol, United Kingdom).

To assess the locus of plasticity expression, we performed coefficient of variation analysis on fEPSP slopes pre-vs. post-HFS. We plotted the CV^2^ ratio (r = CV^2^_(pre)_/CV^2^_(post)_ against the mean slope ratio (m = µ_(post)_/µ_(pre)_ for each experiment. Most points with m > 1 (successful LTP) clustered near or below the identity line (r = 1), indicating a mix of pre- and postsynaptic contributions to LTP.

#### Drugs

GABA_B_R agonist baclofen (Bacl; 20 μM), mGluR1α antagonist LY367385 (LY; 20 μM), mGluR5 antagonist Fenobam (Feno; 10 μM), and GABA_A_R antagonist gabazine (10 μM) were dissolved in dH_2_O or DMSO and sourced from HelloBio (HelloBio Ltd., United Kingdom) or Tocris (Bio-Techne Ltd., United Kingdom).

#### Statistical analysis

All experiments were performed blind to genotype, and data are presented as mean ± SEM. In most cases, one cell or slice was recorded per animal per treatment, limiting the ability to assess intra-animal variability; thus, individual cells or slices were treated as the primary independent replicates. Statistical analyses were conducted on cell-averaged data following assessment of normality. Parametric or non-parametric tests were applied as appropriate, including two-way ANOVA, unpaired Student’s t-tests, Mann–Whitney U-tests, and Wilcoxon signed-rank tests. To determine whether LTP-induction or pharmacological treatment resulted in differences from baseline, 1-sample Wilcoxon signed-rank tests were performed (against 100% baseline), which are indicated on graphs immediately below box-plots. For datasets involving linear regression, comparisons were made using sum-of-squares F-tests. Statistical significance was defined as p < 0.05. Statistical tests and graphing were performed using GraphPad Prism (GraphPad Software v10.4.1, San Diego, CA, United States).

## Results

In the present study, we tested the hypothesis that in *Fmr1* ^-/y^ mice SST-INs display enhanced LTP, consistent with the presence of excessive mGluR1/5 signalling, which leads to impairments in temporoammonic synaptic plasticity. For this, we performed whole-cell and extracellular field recordings from CA1 of adult *Fmr1* ^-/y^ mice, compared to wild-type (WT) littermates.

### CA1 SST-INs show enhanced LTP of TA inputs in *Fmr1* ^*-/y*^ mice

**FIGURE 1:**
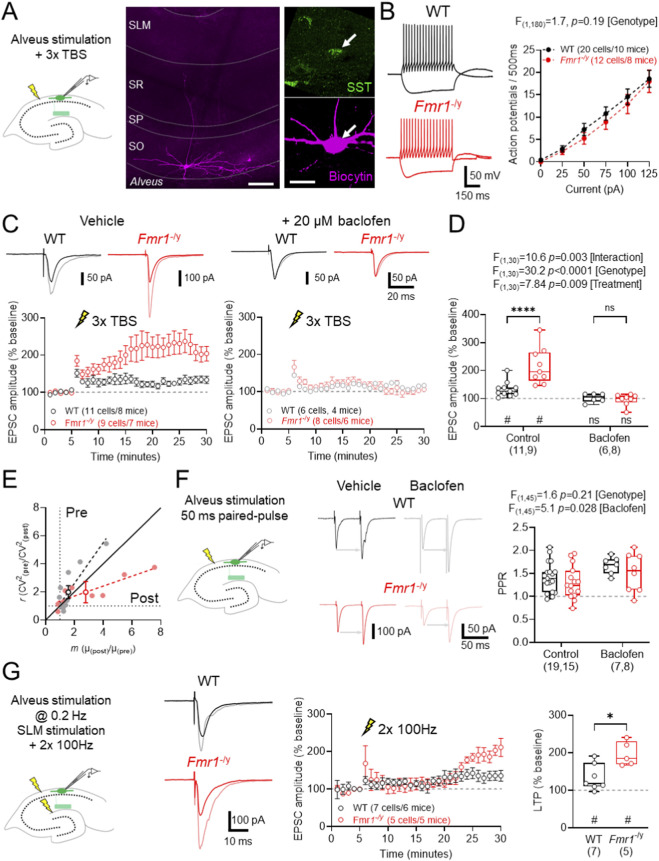
Elevated LTP in CA1 SST-INs in *Fmr1* ^*-/y*^ mice is susceptible to GABA_B_R activation. **(A)** Overview of experiments and identity of recorded cells. Left, schema of recording configuration for measuring aTBS induced LTP in SST-INs. Middle, low magnification flattened confocal stack showing a recorded SST-IN (scale: 100 μm). Right, high magnification image of the same recorded cell (magenta and arrow) confirming SST immunoreactivity (green; scale: 20 μm). **(B)** Example voltage responses of SST-INs from WT (black, upper) and *Fmr1* ^*-/y*^ (red, lower) mice, following ± 125 pA (500 ms duration) stimulation. The average action potential output of all recorded SST-INs is quantified for all current steps delivered. **(C)** Upper, example traces from WT (black) and *Fmr1* ^*-/y*^ (red) mice before and after (grey and pink, respectively) induction of aTBS LTP under vehicle conditions or following 20 min pre-application of 20 μM R-baclofen. Lower, time-course plots of EPSC amplitude all WT (n = 11 cells from 8 mice) and *Fmr1* ^*-/y*^ (n = 9 cells from 7 mice) SST-INs in vehicle and following baclofen pre-treatment (WT: n = 6 cells from 4 mice, grey; *Fmr1* ^*-/y*^: n = 8 cells from 6 mice, pink). **(D)** Comparison of the magnitude of EPSC potentiation measured under control conditions and following baclofen pre-treatment in WT and *Fmr1* ^*-/y*^ SST-INs. **(E)** CV^2^ analysis of aTBS LTP recordings from WT (grey circles) and *Fmr1* ^*-/y*^ (pink circles) SST-INs. The average responses of both genotypes are depicted ±SEM (black and red, respectively) as well as the linear regression of each group (dashed lines). **(F)** Paired-pulse recordings from SST-INs showing stimulation location. Example paired-pulse responses in WT (upper, black) and *Fmr1* ^*-/y*^ (lower, red), in vehicle or 20 μM baclofen. Measured PPR from SST-INs following alveus stimulation. **(G)** Schematic of TA LTP recording, while measuring alveus EPSCs in SST-INs showing stimulation locations. Example alveus EPSCs from WT (upper, black) and *Fmr1* ^*-/y*^ (lower, red), before and after (grey and pink) 2 × 100 Hz stimulation to the SLM. Timecourse of alveus EPSC amplitude following TA LTP induction. Quantification of peak EPSC change 20–25 min post HFS. Data is shown as mean ± SEM **(B,C,E)** or box-plots depicting 25%–75% range, maximum range **(D,F,G)** and individual data points (open circles). Statistics shown from 2-way ANOVA **(B,D,F)** Mann-Whitney **(G)** or Wilcoxon signed-rank **(D,G)** tests, ns-p > 0.05, **** - p < 0.0001 from Holm-Sidak tests; or ns p > 0.05, # - p < 0.05 (Wilcoxon signed-rank test).

SST-INs were targeted for recording at the *str. oriens*/alveus border. In the present study LTP was recorded from 47 cells which met inclusion criteria, of which 38 were recovered for immunohistochemistry. 2 cells were excluded from further analysis as they lacked SST immunoreactivity. SST-INs typically displayed a horizontally-oriented somatodendritic axis, with axons that extended in SLM (Figure 1A). Immediately following achieving whole-cell configuration we measured basal intrinsic excitability of SST-INs in *str. oriens* of CA1. In response to hyperpolarising current injections SST-INs produced large amplitude sag potentials, consistent with a high I_h_ of these cells, while depolarisation consistently gave rise to medium-fast action potential discharge in both WT and *Fmr1* ^-/y^ mice. No difference in action potential output was observed between genotypes (Figure 1B).

Associative theta-burst stimuli (aTBS) induced LTP in SST-INs is known to be mediated by group 1 mGluRs (Topolnik et al., 2006), and which is sensitive to GABA_B_R activation (Booker et al., 2018). To determine whether this form of synaptic plasticity was altered in *Fmr1* ^-/y^ mice, we stimulated the alveus and recorded EPSCs from SST-INs under control conditions and following pre-treatment of slices with the selective agonist R-baclofen (20 µM) for 20 min (Figure 1C). In *Fmr1* ^-/y^ SST-INs, aTBS of alveus inputs induced LTP had an average potentiation at 25–30 min, which was larger than for WT mice (WT: 133.2% ± 8.2% vs. *Fmr1* ^*-/y*^: 216.1% ± 21.7%; t_(11,9)_ = 7.4, p < 0.0001, Holm-Sidak test), indicating enhanced synaptic potentiation in *Fmr1* ^*-/y*^ SST-INs. Baclofen inhibited SST-IN LTP in both WT and *Fmr1* ^-/y^ mice, as the same aTBS induction of SST-INs failed to produce facilitation of EPSCs (WT: 102.5% ± 6.3%; *Fmr1* ^*-/y*^: 96.3% ± 7.4%), which did not differ between genotypes (t_(6,8)_ = 0.29, *p* = 0.77, Holm-Sidak test, Figure 1D). To probe the locus of LTP magnitude differences, we performed squared coefficient of variation analysis (Faber and Korn, 1991). Plotting the SST-IN LTP data from WT mice revealed a relationship close to unity, meanwhile in *Fmr1* ^-/y^ mice this relationship tended to be skewed into the post-synaptic domain (F = 18.9, p = 0.0004, sum of least-squares F-test, Figure 1E). To determine whether differences in LTP were due to altered presynaptic function, we next measured paired-pulse ratio (PPR) of alveus inputs to SST-INs. We found that under control conditions, EPSCs driven by alveus stimulation were similarly facilitating in both WT and *Fmr1* ^-/y^ SST-INs, with pre-application of baclofen leading to elevated PPR in a genotype-independent manner (Figure 1F).

Finally, SST-INs have been shown to undergo long-term strengthening following activation of distal synaptic inputs, which propagate through the local microcircuit (Maccaferri and McBain, 1995). To confirm whether such inputs to CA1 undergo plasticity in our hands, we performed whole-cell recordings from SST-INs whilst stimulating alveus inputs, followed by 2 × 100 Hz stimulation of SLM. We observed robust potentiation of alveus EPSCs 20–25 min following TA stimulation in WT (134.1% ± 13.2% of baseline, t_(6)_ = 2.6, *p* = 0.04, paired t-test) and *Fmr1* ^-/y^ (196.6% ± 14.5% of baseline, t_(4)_ = 3.5, *p* = 0.03, paired t-test) SST-INs, which was higher in the latter (U_(7,5)_ = 5, *p* = 0.048, Mann-Whitney test, Figure 1G).

These data show that although intrinsic excitability of SST-INs is unchanged, excitatory inputs to SST-INs undergo exaggerated LTP in *Fmr1* ^*-/y*^ mice, which is fully supressed by baclofen. This heightened plasticity is likely postsynaptic in origin, propagates through CA1 PNs to SST-INs, and thus could lead to impaired TA plasticity.

### TA–CA1 LTP is maintained in *Fmr1* ^*-/y*^ mice following high-frequency stimulation

**FIGURE 2:**
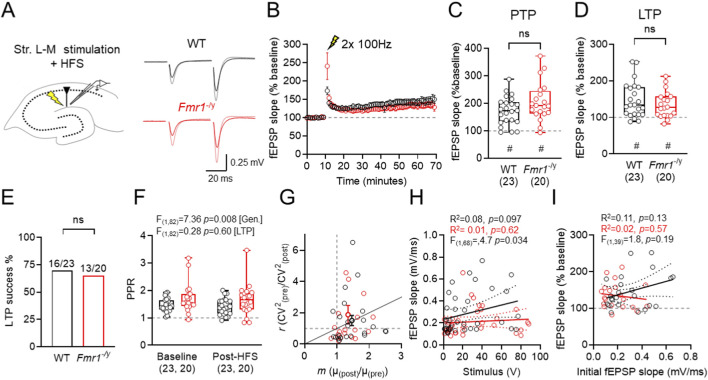
Extracellular field recordings in SLM show intact LTP in the TA–CA1 pathway of *Fmr1* ^*-/y*^ mice. **(A)** Schematic of fEPSP recording configuration, showing recording and stimulation locations, and example fEPSP traces from WT and *Fmr1* ^*-/y*^ mice, recorded before and after HFS (grey and pink, respectively). **(B)** Time-course of fEPSP recordings (compared to baseline) following high-frequency stimulation (HFS, lightning bolt) of TA inputs in CA1 SLM of WT (black) and *Fmr1* ^*-/y*^ mice (red). **(C)** fEPSP slopes measured 50–60 min post-HFS, relative to baseline. **(D)** Proportion of successful LTP inductions, defined as fEPSP slope change >10% above baseline 50–60 min post-HFS. **(E)** Post-tetanic potentiation (PTP) magnitude measured 1 min after HFS. **(F)** PPR recorded before (baseline) and post-HFS. **(G)** Coefficient-of-variation squared (CV^2^) analysis of pre- and post-HFS responses. **(H)** Comparison of stimulus strength vs. fEPSP slope for all fEPSP recordings. **(I)** Comparison of baseline fEPSP slope and fEPSP potentiation at 50–60 min post-HFS. All data shown from WT: n = 23 slices; *Fmr1* ^*-/y*^: n = 20 slices. Data is shown as mean ± SEM **(B)** or box-plots depicting 25%–75% range, maximum range **(C,E,F)** or as scatter plots **(G,H,I)** all with individual data points overlaid (open circles). Statistics shown from Mann-Whitney tests **(C,E)** 2-way ANOVA **(F)** linear regression with F-tests **(H,I)** and or Wilcoxon signed-rank **(C,E)** tests. Statistical significance shown as: ns-p > 0.05; or ns p > 0.05, # - p < 0.05 (Wilcoxon signed-rank test).

Given that SST-IN LTP is elevated in *Fmr1* ^-/y^ mice, we next asked if this led to impaired LTP at TA inputs in SLM of CA1. To assess this we recorded field excitatory postsynaptic potentials (fEPSPs) from the SLM of CA1, which reflects the response of TA inputs onto PN distal dendrites (Figure 2A). Synaptic strength was monitored as fEPSP slope during a 10 min baseline, then a high-frequency stimulation (HFS; 2 × 1 s, 100 Hz) was delivered to induce LTP (Figure 2B). In CA1 of WT mice (23 slices) we observed robust post-tetanic potentiation (PTP) immediately (1–2 min) following HFS to the SLM 173.2% ± 10.6% (p < 0.001, Wilcoxon signed rank test, Figure 2C). When measured at 50–60 min post-HFS, we observed increased fEPSP slopes, relatively to baseline of 146.5% ± 10.0% (p < 0.0001, Wilcoxon signed rank test, Figure 2D). Based on a criterion of 10% facilitation at 50–60 min post HFS constituting LTP, 16 of 23 slices displayed LTP in WT mice. Similarly, in *Fmr1* ^-/y^ mice (20 slices), we observed PTP of 206.4% ± 15.7% (p < 0.0001, Wilcoxon signed-rank test) which did not differ from WT slices (U_(42)_ = 172, *p* = 0.163; Mann-Whitney test, Figure 2C). Likewise, fEPSP slopes at 50–60 min post HFS were elevated to 134.5% ± 8.0% (*p* = 0.0002, Wilcoxon signed-rank test), which did not differ between genotypes in terms of magnitude (U_(42)_ = 200, *p* = 0.476; Mann-Whitney test, Figure 2D). 13 of 20 *Fmr1* ^*-/y*^ slices displayed successful LTP inductions, which was not different from WT slices (p = 0.75, Chi-square test, Figure 2E).

Despite no difference in LTP, we did observe genotype-dependent differences in baseline synaptic transmission and short-term plasticity. In particular, the paired pulse ratio (PPR) of pre-HFS fEPSP was significantly higher in *Fmr1* ^*-/y*^ slices compared to WT, independent of LTP induction (F_(1,82)_ = 7.36; [*genotype*], *p* = 0.008; Two-way ANOVA; Figure 2F), suggesting a FMRP dependent effect on release probability; despite no difference in CV^2^ analysis (Figure 2G). Consistent with our previous observations (Booker et al., 2020), synaptic strength was weaker at TA inputs to CA1, as reflected by a lower y-intercept of *Fmr1* ^-/y^ slices when fEPSP slope vs. stimulation intensity is plotted (F_(1,68)_ = 4.704; *p* = 0.034; [*intercepts*]; Figure 2H). We did not find any genotype difference in the magnitude of LTP compared to the initial fEPSP slope (F_(1,39)_ = 1.8; *p* = 0.19; [*slope*]; Figure 2I).

Together, these data suggest that although *Fmr1* ^*-/y*^ mice exhibit reduced basal synaptic function (higher PPR, lower fEPSP slopes) at TA inputs to CA1, overall LTP magnitude is maintained. The preservation of TA-CA1 LTP in Fmr1^-/y^ slices despite heightened SST-IN plasticity may reflect compensatory effects, such as impaired inhibition.

### Pharmacological modulation of GABA_B_Rs, GABA_A_Rs and group 1 mGluRs minimally affects basal synaptic transmission at TA inputs

**FIGURE 3:**
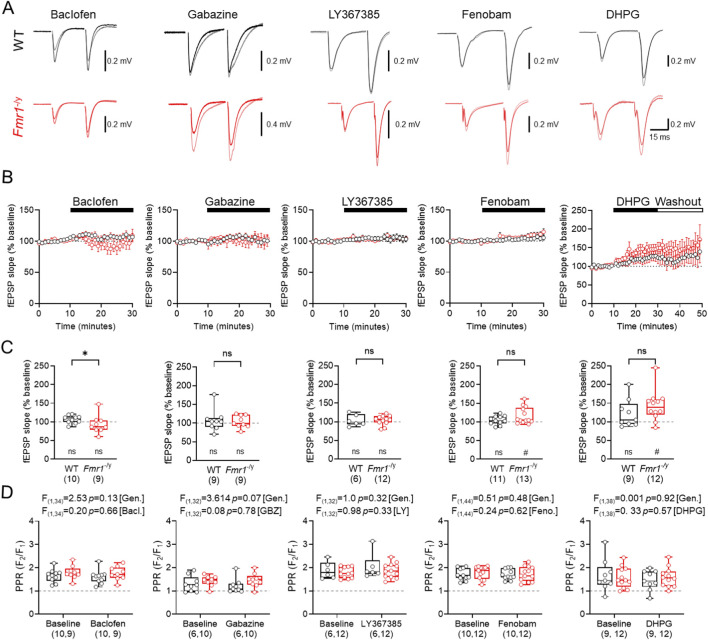
Comparison of GABA and mGluR modifying drugs on TA basal synaptic transmission **(A)** Example fEPSP traces recorded before and after drug incubation in WT (black) and *Fmr1* ^*-/y*^ (red) mice. Drugs applied from left to right include: 20 μM baclofen (GABA_B_R agonist), 10 μM gabazine (GABA_A_R antagonist), 20 μM LY367385 (mGluR1α antagonist), 10 μM Fenobam (mGluR5 antagonist), and 10 μM s-DHPG (mGluR1/5 agonist). **(B)** Timecourse plots shown separately for each treatment condition and genotype. The duration of each drug application is shown above (black bar). **(C)** Mean fEPSP slope measured over the final 10 min of drug application for each of the drugs listed. **(D)** Paired-pulse ratio (PPR) before and after drug application. Data shown as mean ± SEM **(B)** or box-plots depicting 25%–75% range, maximum range **(C,D)**. WT and *Fmr1* ^*-/y*^ sample sizes indicated in each bar); all with individual data points overlaid (open circles). Statistics shown from Mann Whitney tests **(C)** 2-way ANOVA **(D)** and Wilcoxon signed-rank tests **(C)**. Statistics shown as: ns-p > 0.05, * - p < 0.05; or ns p > 0.05, # - p < 0.05 (Wilcoxon signed-rank test).

As the inhibitory potential of SST-INs is regulated by GABA_A_R, GABA_B_Rs, and mGluRs (Topolnik et al., 2006; Ali and Thomson, 1998; Leão et al., 2012; Booker et al., 2018) we next determined the effect that modulating these receptors had on basal fEPSPs evoked by TA stimulation in WT and *Fmr1* ^-/y^ mice. To achive this, we bath applied GABA_B_R agonist baclofen (20 μM), GABA_A_R antagonist gabazine (SR95531, 10 μM), mGluR1α antagonist LY367385 (20 μM), mGluR5 negative allosteric modulator Fenobam (10 μM), and the mGluR1/5 agonist s-DHPG (10 μM) to paired fEPSPs (50 ms interval) evoked by TA stimulation (Figure 3A). Measurement of the fEPSP slope during wash-in resulted in minimal observable difference in overal TA response during the 20 min wash-in period for baclofen (WT n = 10; *Fmr1* ^*-/y*^ n = 9 slices), gabazine (WT n = 9; *Fmr1* ^*-/y*^ n = 9 slices), and LY367385 (WT n = 6; *Fmr1* ^*-/y*^ n = 12 slices) (Figure 3B). However, Fenobam (WT n = 11; *Fmr1* ^*-/y*^ n = 13 slices) and DHPG (WT n = 9; *Fmr1* ^*-/y*^ n = 9 slices) both led to an increase in fEPSP slopes in *Fmr1* ^-/y^ slices. Comparing whether these drugs displayed genotype-specific differences in activity, we found that fEPSP slopes in *Fmr1* ^-/y^ slices following baclofen wash-in were signifcantly lower than WT (U_(18)_ = 19, *p* = 0.035, Mann-Whitney test). However, we found that gabazine (U_(17)_ = 39, *p* = 0.93, Mann-Whitney test), LY367385 (U_(17)_ = 33, *p* = 0.82, Mann-Whitney test), Fenobam (U_(23)_ = 53, *p* = 0.30, Mann-Whitney test), nor DHPG (U_(20)_ = 36, *p* = 0.22, Mann-Whitney test), did not diplay genotype-specific differences in fEPSP slope (Figure 3C). Equally, none of these drugs signficantly altered PPR at TA inputs to CA1 (Figure 3D). Together, these data reveal that while GABA_B_Rs may display some genotype effects, there is minimal difference in mGluR1/5 and GABA_A_R mediated control of TA inputs to CA1.

### GABA receptor modulation does not differentially effect TA LTP in WT and *Fmr1* ^*-/y*^ mice

**FIGURE 4:**
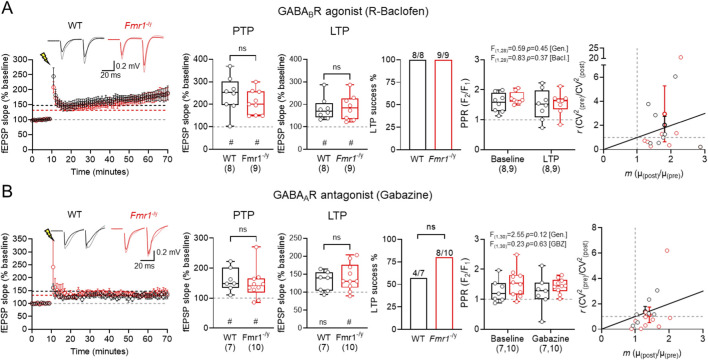
No genotype-specific effects of GABA modulating drugs on TA LTP in *Fmr1* ^*-/y*^ mice. **(A)** Time-course of fEPSP slope following HFS (lightning bolt) at TA inputs in wild-type (WT, black) and *Fmr1* ^*-/y*^ (red) mice, following 30 min incubation with 20 μM baclofen followed by a 20 min washout. Inset, example fEPSP traces from WT and *Fmr1* ^*-/y*^ mice recorded before (black and red, respectively) and after HFS (grey and pink, respectively). PTP magnitude measured 1 min after HFS and LTP measured 50–60 min post-HFS, both relative to baseline fEPSP slope. Proportion of mice exhibiting successful LTP induction, defined as a 50–60 min post-HFS slope >10% above baseline. PPR measured before and after HFS, and CV^2^ analysis pre- and post-HFS responses. WT: n = 8 slices; *Fmr1* ^*-/y*^: n = 9 slices. **(B)** The same data, but shown for TA LTP recordings performed after 30 min incubation with the GABA_A_R antagonist gabazine (10 μM). WT: n = 7 mice; *Fmr1* ^*-/y*^: n = 10 mice. Data is shown as mean ± SEM or box-plots depicting 25%–75% range, maximum range; all with individual data points overlaid (open circles). Statistics shown from Mann-Whitney tests, Chi-squared tests, 2-way ANOVA or Wilcoxon signed-rank tests; ns-p > 0.05 (Mann-Whitney test); or ns p > 0.05, # - p < 0.05 (Wilcoxon signed-rank tests).

SST-IN activation recruits both GABA_A_Rs and GABA_B_Rs (Watson and Booker, 2024), and their plasticity itself is regulated by GABA_B_R activation (Booker et al., 2018). As such, we next determined whether GABA_B_R activation (Figure 4A), or GABA_A_R inhibition (Figure 4B) differentially regulates TA LTP. Following baclofen (20 uM) application we induced TA LTP as before. Following 2 × 100 Hz HFS, we observed robust PTP in WT of 244.4% ± 28.5% (n = 8 slices, *p* = 0.0078, Wilcoxon signed-rank test) and 208.7% ± 18.2% in *Fmr1* ^-/y^ (n = 9 slices, *p* = 0.0039, Wilcoxon signed-rank test), which was not different between genotypes (U_(16)_ = 26, *p* = 0.37, Mann-Whitney test). Consistently, at 50–60 min post HFS, we observed robust potentiation of fEPSP slopes in WT of 181.1% ± 17.6% (n = 8 slices, *p* = 0.0078, Wilcoxon signed-rank test) and 186.8% ± 20.0% in *Fmr1* ^-/y^ (n = 9 slices, *p* = 0.0078, Wilcoxon signed-rank test), which again was not different between genotypes (U_(16)_ = 30.5, *p* = 0.90, Mann-Whitney test). A direct within-genotype comparison of the effect of baclofen is shown in Figure 6. Following baclofen application, all slices from both genotypes displayed LTP induction (WT: 8/8, *Fmr1* ^*-/y*^: 9/9). We found no genotype or LTP-dependent effects on PPR in WT or *Fmr1* ^*-/y*^ slices (F_(1,28)_ = 0.59, *p* = 0.45; [*genotype*]; F_(1,28)_ = 0.83; *p* = 0.37; [HFS]; Two-way ANOVA). CV^2^ analysis revealed both pre- and postsynaptic mechanisms contributing to LTP in both genotypes in slices treated with baclofen.

Following application of gabazine (Figure 4B) we observed robust PTP in WT of 161.2% ± 14.7% (n = 7 slices, *p* = 0.0156, Wilcoxon signed-rank test) and 147.4% ± 16.1% of *Fmr1* ^*-/y*^ (n = 10 slices, *p* < 0.01, Wilcoxon signed-rank test), with no differences between genotype (U_(16)_ = 26, *p* = 0.42, Mann-Whitney test). At 50–60 min post-HFS we did not observe significant LTP in WT of 129.3% ± 10.6% (n = 7, *p* = 0.078, Wilcoxon signed-rank test) only LTP in *Fmr1* ^*-/y*^ of 141.7% ± 12.0% (n = 10, *p* = 0.0059, Wilcoxon signed-rank test), which was not different between genotypes (U_(16)_ = 31, *p* = 0.74, Mann-Whitney test). A direct within-genotype comparison of the effect of gabazine is shown in Figure 6. In the presence of gabazine, successful LTP induction was not different from control (WT: 4/7, *Fmr1* ^*-/y*^: 8/10). We found no genotype or LTP dependent effect on PPR (F_(1,30)_ = 2.55, *p* = 0.12; [*genotype*]; F_(1,30)_ = 0.23, *p* = 0.63, [*HFS*], Two-way ANOVA). CV^2^ analysis suggested that gabazine induced a predominantly postsynaptic-mediated induction of LTP.

These data indicate that there are no genotype specific effects of GABA_A_R inhibition or GABA_B_R activation on TA LTP in CA1, in the *Fmr1* ^-/y^ mouse. This indicates that perhaps enhanced SST-IN LTP has a limited net effect on this pathway.

### mGluR modulation also does not differentially effect TA LTP in WT and *Fmr1* ^*-/y*^ mice

LTP in SST-INs requires activation of mGluR1α and mGluR5 (Topolnik et al., 2006; Vasuta et al., 2015), and indeed mGluR1/5 have been implicated in altered hippocampal plasticity of *Fmr1* ^-/y^ mice (Osterweil et al., 2010). Thus, we next determined the effects of blocking mGluR1α, inhibiting mGluR5, or activating both of these receptors on TA LTP.

**FIGURE 5:**
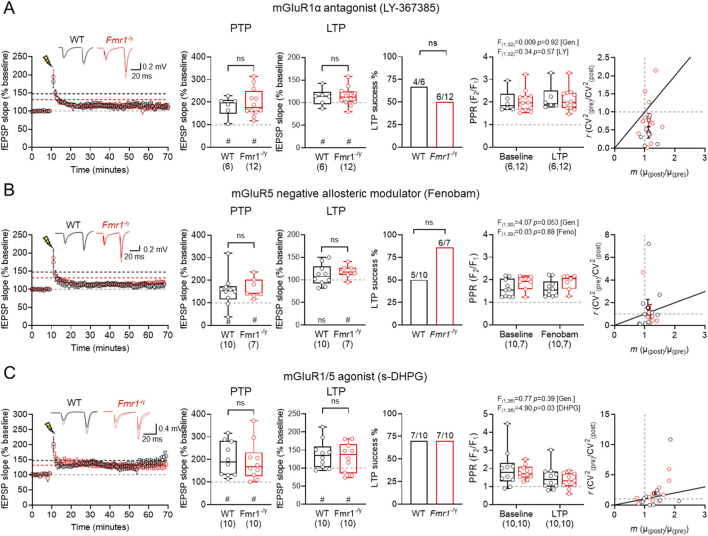
Group 1 mGluR modulation has no genotype effects on TA LTP in *Fmr1* ^*-/y*^ mice. **(A)** Time-course of fEPSP slope following HFS at TA inputs in wild-type (WT, black) and *Fmr1* ^*-/y*^ (red) mice, following 30 min bath application of the mGluR1α antagonist LY367385 (20 μM). Inset, example fEPSP traces from WT and *Fmr1* ^*-/y*^ mice recorded before (black and red, respectively) and after HFS (grey and pink, respectively). PTP magnitude measured 1 min after HFS and LTP measured 50–60 min post-HFS, both relative to baseline fEPSP slope. Proportion of mice exhibiting successful LTP induction, defined as a 50–60 min post-HFS slope >10% above baseline. PPR measured before and after HFS, and CV^2^ analysis pre- and post-HFS responses. WT: n = 6 slices; *Fmr1* ^*-/y*^: n = 12 slices. **(B)** The same data, but shown for TA LTP recordings performed after 30 min incubation with the mGluR5 antagonist Fenobam (10 μM). WT: n = 10 mice; *Fmr1* ^*-/y*^: n = 7 mice. **(C)** The same data, but shown for recordings performed after 30 min incubation with the mGluR1/5 agonist s-DHPG (10 μM). WT: n = 10 slices; *Fmr1* ^*-/y*^: n = 10 slices. Data is shown as mean ± SEM or box-plots depicting 25%–75% range, maximum range; all with individual data points overlaid (open circles). Statistics shown from Mann-Whitney tests, Chi-squared tests, 2-way ANOVA or Wilcoxon signed-rank tests; ns-p > 0.05 (Mann-Whitney test); or ns p > 0.05, # - p < 0.05 (Wilcoxon signed-rank tests).

For mGluR1α, following LY367385 bath-application (Figure 5A), we observed PTP in WT of 182.0% ± 17.8% (n = 6 slices, *p* = 0.031, Wilcoxon signed-rank test) and *Fmr1* ^*-/y*^ slices of 198.5% ± 17.9% (n = 12 slices, *p* < 0.001, Wilcoxon signed-rank test), with no significant difference between genotypes (U_(17)_ = 34, *p* = 0.89, Mann-Whitney test). At 50–60 min post-HFS, LTP was not observed in WT of 112.5% ± 7.6% (n = 6 slices, *p* = 0.09, Wilcoxon signed-rank test) or *Fmr1* ^*-/y*^ slices of 113.7% ± 5.9% (n = 12 slices, *p* = 0.034, Wilcoxon signed-rank test), and no difference between genotype (U_(17)_ = 33, *p* = 0.82, Mann-Whitney test). WT and *Fmr1* ^*-/y*^ slices showed reduced, but comparable LTP success (WT: 4/6, *Fmr1* ^*-/y*^: 6/12; *p* = 0.50, Chi-square test). LY367385 did not significantly alter PPR post-HFS (F_(1,32)_ = 0.34; *p* = 0.57; [*LY367385*]; Two-way ANOVA) in either WT (Pre: 2.02 ± 0.2 vs. Post: 2.17 ± 0.23) or *Fmr1* ^*-/y*^ (Pre: 2.05 ± 0.14 vs. Post: 2.11 ± 0.14; F_(1,32)_ = 0.01; *p* = 0.92; [*genotype*]). CV^2^ analysis revealed that the minimal LTP induced in the presence of LY367385 was predominantly postsynaptic in nature.

In slices incubated with Fenobam (Figure 5B) we observed PTP in WT of 155.3% ± 22.7% (n = 10 slices, *p* = 0.49, Wilcoxon signed-rank test) and *Fmr1* ^*-/y*^ PTP of 164.7% ± 16.5% was similar (n = 7 slices, *p* = 0.016, Wilcoxon signed-rank test), with no significant genotype effects (U_(16)_ = 33, *p* = 0.89, Mann-Whitney test). We found Fenobam to consistently reduce LTP magnitude in WT slices of 110.6% ± 7.2% (n = 10 slices, *p* = 0.32, Wilcoxon signed-rank test) while *Fmr1* ^*-/y*^ slices continued to display LTP of 118.6% ± 5.4% (n = 7 slices, *p* = 0.03, Wilcoxon signed-rank test) with no significant difference between genotype (U_(16)_ = 24, *p* = 0.32, Mann-Whitney test). The LTP success rate following Fenobam application was similar between WT and *Fmr1* ^*-/y*^ slices (WT: 5/10 vs. *Fmr1* ^*-/y*^: 6/7; p = 0.13, Chi-square test). Fenobam did not alter PPR post-HFS with no main effect of treatment (F_(1,30)_ = 0.0253; *p* = 0.88; [Fenobam]; Two-way ANOVA). We observed a tendency for PPR to be lower post-HFS in WT (Pre: 1.62 ± 0.12 vs. Post: 1.63 ± 0.12) compared to *Fmr1* ^*-/y*^ (Pre: 1.86 ± 0.13 vs. Post: 1.90 ± 0.13; F_(1,30)_ = 4.065; [*genotype*], *p* = 0.053). CV^2^ analysis revealed a predominantly postsynaptic locus.

In slices incubated with 10 μM DHPG (Figure 5C), a concentration known to induce LTP at unitary inputs onto SST INs (Le Vasseur et al., 2008), we observed PTP in WT of 201.9% ± 24.0% (n = 10 slices, *p* = 0.002, Wilcoxon signed-rank test) and *Fmr1* ^*-/y*^ slices of 191.6% ± 26.9% (n = 10 slices, *p* = 0.002, Wilcoxon signed-rank test), with no significant genotype effects (U_(19)_ = 45, *p* = 0.74, Mann-Whitney test). Following DHPG application we found HFS to consistently induce robust LTP in WT slices of 137.6% ± 12.2% (n = 10 slices, *p* = 0.014, Wilcoxon signed-rank test) and *Fmr1* ^*-/y*^ slices of 130.3% ± 12.7% (n = 10 slices, *p* = 0.048, Wilcoxon signed-rank test) with no significant difference between genotype (U_(19)_ = 45, *p* = 0.74, Mann-Whitney test). The LTP success rate following DHPG application was similar between WT and *Fmr1* ^*-/y*^ slices (WT: 7/10 vs. *Fmr1* ^*-/y*^: 7/10; p = 0.99, Chi-square test). DHPG on PPR revealed a significant effect of treatment (F_(1,36)_ = 4.899; *p* = 0.033; [DHPG]; Two-way ANOVA). We observed a tendency PPR to be lower post-HFS in WT (Pre: 1.98 ± 0.33 vs. Post: 1.49 ± 0.21) and *Fmr1* ^*-/y*^ slices (Pre: 1.78 ± 0.12 vs. Post: 1.31 ± 0.13; F_(1,36)_ = 0.7702; [*genotype*], *p* = 0.39). CV^2^ analysis revealed both pre- and postsynaptic mechanisms contributing to LTP in both genotypes in the presence of DHPG.

Taken together, these data indicate that Group 1 mGluRs, which are known to contribute to SST-IN plasticity, do not differentially effect TA LTP in *Fmr1* ^-/y^ mice; rather these drugs modulate LTP in a consistent manner between genotypes. This implies that enhanced SST-IN LTP in *Fmr1* ^*-/y*^ mice either does not propagate to circuit level, or that effects are nuanced with broad pharmacological approaches saturating effects.

### Comparison of different modifiers of GABA and group1 mGluR signalling

**FIGURE 6:**
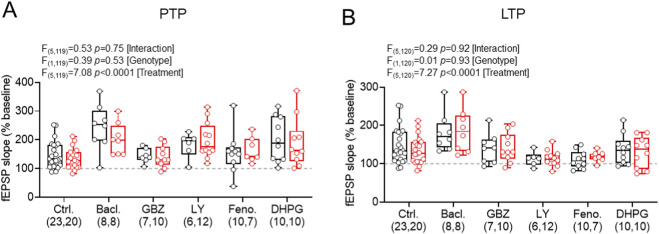
Summary of drug incubations on PTP and LTP magnitude post-HFS revealed no significant differences. **(A)** Post-HFS fEPSP slope (measured 0–1 min after stimulation) for PTP measured under control conditions and following application of baclofen (GABA_B_R agonist), gabazine (GABA_A_R antagonist), LY367385 (mGluR1α antagonist), Fenobam (mGluR5 antagonist), or s-DHPG (mGluR1/5 agonist); in wild-type (WT, black) and *Fmr1* ^*-/y*^ (red) mice. **(B)** Data in the same form, but shown for LTP measured at 50–60 min post-HFS. All data shown as box-plots depicting 25%–75% range, maximum range; all with individual data points overlaid (open circles). Sample sizes (n = slices per group) are shown underneath. Statistics shown from 2-way ANOVA.

To allow direct comparison between fEPSP recordings made under control conditions and following drug application, we performed a side-by-side comparison of PTP and LTP effects (Figure 6).

For PTP (Figure 6A), groupwise comparison revealed a significant effect of treatment (F_(5,119)_ = 7.08; *p* < 0.0001 [treatment]; 2-way ANOVA). In WT TA recordings, we observed that baclofen (t_(23,8)_ = 4.14, p < 0.0001, Fishers LSD test) and DHPG (t_(23,10)_ = 2.43, p = 0.017, Fishers LSD test) significantly increased PTP, but gabazine (t_(23,7)_ = 0.14, p = 0.89, Fishers LSD test), LY367385 (t_(23,6)_ = 1.32, p = 0.19, Fishers LSD test), and Fenobam (t_(23,10)_ = 0.36, p = 0.72, Fishers LSD test) did not. We found no effect of genotype (F_(1,119)_ = 0.39; *p =* 0.53; 2-way ANOVA), or interaction of genotype and treatment (F_(5,119)_ = 0.53; *p =* 0.75; 2-way ANOVA). These indicate that modulators of GABA and mGluRs appear to display differential effects on presynaptic-induced short-term plasticity in a genotype independent manner.

Comparing LTP outcomes (Figure 6B), groupwise comparison also revealed a significant treatment effect (F_(5,120)_ = 7.27; *p* < 0.0001; 2-way ANOVA). In WT TA LTP recordings, we observed that baclofen treatment increased LTP (t_(23,8)_ = 2.13, p = 0.035, Fishers LSD test), but LY367385 (t_(23,6)_ = 1.99, p = 0.049, Fishers LSD test) and Fenobam (t_(23,10)_ = 2.54, p = 0.012, Fishers LSD test) both substantially decreased LTP. Meanwhile, gabazine (t_(23,8)_ = 0.48, p = 0.63, Fishers LSD test) and DHPG (t_(23,10)_ = 0.68, p = 0.50, Fishers LSD test) had no apparent effect on TA LTP. Again, we found no effect of genotype (F_(1,120)_ = 0.01; *p =* 0.93; 2-way ANOVA), or interaction of genotype and treatment (F_(5,120)_ = 0.29; *p =* 0.92; 2-way ANOVA).

Together, these data confirm our earlier findings of an absence of genotype effect on TA LTP in *Fmr1* ^-/y^ mice. Furthermore, baclofen, LY367385, and Fenobam (which are all predicted to block SST-IN LTP) all modified LTP, albeit not in the same direction. These findings confirm the potential role of SST-INs in TA LTP, but that its induction and maintenance is not impaired in *Fmr1* ^-/y^ mice.

## Discussion

Our findings demonstrate that SST-INs play a significant role in shaping LTP in the TA pathway, but that while they themselves display enhanced LTP, their control of plasticity at this pathway is not altered in a mouse model of FXS. This dissociation suggests that hippocampal circuits in *Fmr1* ^-/y^ mice likely compensate for reduced TA input and elevate SST-IN LTP to maintain a functional transfer of spatial information.

We found no significant change in the intrinsic excitability of SST-INs in adult *Fmr1* ^*-/y*^ mice. This is notable, given the broader neuronal hyperexcitability reported in CA1 PNs (Booker et al., 2020; Bataveljic et al., 2024; Luque et al., 2017; Luque et al., 2024), as well as in the neocortex of *Fmr1* ^*-/y*^ mice (Deng and Klyachko, 2016). One plausible explanation is that homeostatic plasticity normalises SST-IN excitability over development. Alternatively, FMRP-related ion channel changes (e.g., in HCN or Kv channels) might be offset by other compensation in SST-INs (Hewitt et al., 2025), however we did not observe the same hypoexcitability of SST-INs that Hewitt and colleagues report. Regardless, the major changes in SST-IN function in models of FXS appear at the synaptic level rather than in inherent excitability. Indeed, we observed a robust enhancement of LTP at excitatory synapses onto SST-INs in *Fmr1* ^*-/y*^ mice. While WT SST-INs displayed modest potentiation following aTBS, *Fmr1* ^*-/y*^ SST-INs exhibited far greater potentiation. This is consistent with excessive activation of mGluRs, as has been reported previously for FXS (Osterweil et al., 2010), and which are required for LTP of SST-INs (Topolnik et al., 2006; Le Vasseur et al., 2008). Furthermore, we confirm that activation of CA1 PNs via the TA path leads to propagation of excessive LTP onto SST-INs in the *Fmr1* ^-/y^ mouse, as previously described in WT mice (Maccaferri and McBain, 1995) providing validation of our fEPSP experiments. Together, this suggests that loss of FMRP leads to upregulated plasticity of excitatory inputs onto SST-INs.

**FIGURE 7:**
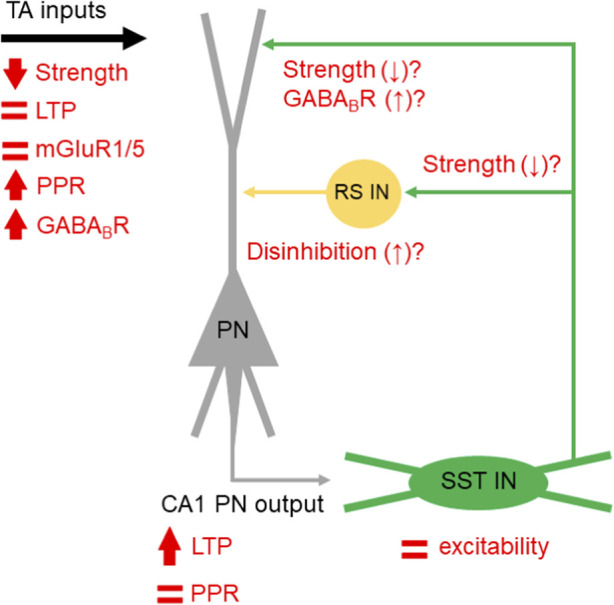
Summary of proposed and speculative changes in synaptic function in the SST-IN microcircuit in CA1. A schematic PN (grey) and SST-IN (green) are shown, with observed function in *Fmr1* ^*-/y*^ mice indicated (red symbols).

What are the circuit-level consequences of exaggerated SST-IN LTP? Our data suggests that such plasticity fails to supress TA LTP in *Fmr1* ^-/y^ mice, when measured with fEPSPs. This is contrary to recent reports when all inhibition (both GABA_A_Rs and GABA_B_Rs) was blocked (Ordemann et al., 2021). In our study, we have not blocked GABA_B_R signalling, specifically as it has been shown to modify CA1 inputs via presynaptic mechanisms in *Fmr1* ^-/y^ mice (Wahlstrom-Helgren and Klyachko, 2015). The fact that we see no differential effect of baclofen application may reflect as saturation of such presynaptic inhibition, as SST-IN input and output is strongly regulated by GABA_B_R activation (Booker et al., 2020). This suggests a more complex picture, where TA inputs to CA1 are reduced in amplitude, undergo typical plasticity mechanisms under pharmacologically naïve conditions, possess elevated GABA_B_R presynaptic function, combined with excessive SST-IN LTP. The most parsimonious explanation for these various effects is a compensatory reduction in the output of SST-INs (Figure 7). However, reduced SST-IN synaptic strength alone would not explain the typical expression of TA LTP we observe in *Fmr1* ^-/y^ mice, as these interneurons are known to inhibit dendritic plasticity mechanisms in CA1 PNs (Udakis et al., 2025). Such effects could require enhanced disinhibitory mechanisms known to result from SST-IN recruitment (Leão et al., 2012; Vasuta et al., 2015), and vasoactive intestinal peptide (VIP) -expressing INs (which mostly target other INs) display elevated activity in *Fmr1* ^-/y^ mice (Rahmatullah et al., 2023). Such a compensatory rebalancing of inhibitory microcircuits may come at the expense of network fidelity, where poor signal-to-noise ratios or disrupted temporal coding potentially contribute to cognitive inflexibility in FXS (Asiminas et al., 2022). Dissection of these mechanisms remains unexplored, but may hold the key to understanding hippocampal circuit function and plasticity in rodent models of FXS.

Our pharmacological manipulations directly investigated translationally-relevant plasticity mechanisms of hippocampal circuits relating to FXS therapies. In FXS, GABA_B_R signalling shows dysregulation, with presynaptic GABA_B_R-mediated inhibition stronger at inhibitory synapses, reducing GABA release (Wahlstrom-Helgren and Klyachko, 2015), and postsynaptic GABA_B_R signalling downregulated (Kang et al., 2017). Application of R-baclofen in acute slices activates all membrane localised GABA_B_Rs (Lüscher et al., 1997), including those that inhibit SST-INs (Booker et al., 2018; Booker et al., 2020), but also those expressing parvalbumin (Booker et al., 2013), cholecystokinin (Booker et al., 2017), and neurogliaform types (Oláh et al., 2007); as well as hippocampal PNs (Degro et al., 2015). Such motifs of GABA_B_R inhibition of PNs and INs appear to be common to CA1 and the rodent dentate gyrus (Degro et al., 2024), suggesting that common motifs of GABA_B_R inhibition may be found throughout the cortex. How these various GABA_B_R signalling mechanisms interact and contribute to circuit level processing in FXS is not yet clear, however such diversity may explain why facilitating GABA_B_R signalling has benefit to behavioural and network phenotypes in *Fmr1* ^-/y^ mice (Henderson et al., 2012; Sinclair et al., 2017; Zhang et al., 2015) and improved some features affected individuals (Berry-Kravis et al., 2012). GABA_A_Rs also display impaired expression in *Fmr1* ^-/y^ mice (d'Hulst et al., 2006), thus silencing GABA_A_Rs may be predicted to have disproportionate genotype-specific effects. Our data revealed no differential effect of the GABA_A_R antagonist gabazine on TA-LTP in WT mice, in agreement with similar recordings from the Schaffer-collaterals (Chapman et al., 1998). This suggests two possibilities: a) that phasic GABA_A_R-mediated inhibition is not a major constraint on LTP in this pathway, or b) that broad administration of gabazine abolishes both direct inhibition and also disinhibition. The latter option is perhaps most likely, as GABA_B_R activation is also known to enhance LTP through disinhibitory mechanisms (see Kulik et al., 2018).

In contrast to GABA_B_R activation, inhibiting mGluR1α with LY367385 or mGluR5 with Fenobam similarly impaired LTP in both genotypes, which we attribute to the loss of SST-IN LTP, as mGluR1α is highly enriched and required for their LTP induction (Vasuta et al., 2015); despite no change to basal TA transmission. As SST-INs displayed enhanced plasticity in *Fmr1* ^*-/y*^ mice, these data indicate that mGluR1/5 inhibition impairs TA LTP induction independent of genotype. Interestingly, stimulation of mGluR1/5 with DHPG, at a concentration above its EC50 and known to induce LTP in SST INs (Le Vasseur et al., 2008) but not widely used to assess population level LTD., revealed no differential effect on basal synaptic transmission nor TA LTP. This is somewhat at odds with suggested exaggerated function of group 1 mGluRs in *Fmr1* ^-/y^ mice (Osterweil et al., 2010), as we find no evidence for LTD. mechanisms of fEPSPs, conversely we observe increased synaptic strength, which did not differ between genotypes. As FMRP is found throughout the dendrites (Hale et al., 2021), it is perplexing that we did not observe genotype specific differences in DHPG function. Further investigation is required to identify mGluR1/5 function in hippocampal distal dendrites. From a therapeutic perspective, mGluR1α antagonism would have limited therapeutic benefit, as this fully and selectively suppresses SST-IN function and may reduce typical circuit function. Conversely, mGluR5 inhibition (e.g., Fenobam) is consistent with the mGluR theory of FXS (Bear et al., 2004). Our data suggests that Fenobam had limited differential effect on TA LTP in *Fmr1* ^-/y^ mice, which with an absence of DHPG effects, may explain the mixed outcomes of mGluR5 blocker trials in FXS patients (Berry-Kravis et al., 2016; Berry-Kravis et al., 2009; Jacquemont et al., 2011), which addressed some behavioural symptoms but did not markedly improve cognition. Further targeted research is needed to disentangle the functional interaction of disinhibition and mGluR signalling mechanisms at long-range synaptic connections.

### Summary

In summary, our data reveal that despite enhanced LTP of SST-INs, TA LTP is largely typical in a mouse model of FXS. Further, we show that mGluR1α, mGluR5, GABA_B_Rs, and GABA_A_Rs do not display genotype selective effects on TA LTP in *Fmr1* ^-/y^ mice. These results highlight that while cell-type specific effects on synaptic function may exist, these appear to be largely compensated for at the circuit level.

## Data Availability

The raw data supporting the conclusions of this article will be made available by the authors, upon reasonable request.
